# One small step…

**DOI:** 10.1186/s40900-015-0005-8

**Published:** 2015-06-25

**Authors:** Richard Stephens, Sophie Staniszewska

**Affiliations:** 1 Involved and engaged patient, Stevenage, Herts UK; 2 Royal College of Nursing Research Institute, University of Warwick, Coventry, England

## Abstract

This editorial introduces the new, online, open-access journal *Research Involvement and Engagement*. The journal considers manuscripts on any aspect of the engagement or involvement of patients, carers or members of the public in design, delivery and dissemination of research in health or social care.

We support the involvement of patients in the production of the journal, as co-authors, as reviewers and as consumers of the journal, through plain English summaries. We want patients and the wider public to become engaged with research and influence its future direction. Through the co-production of the Journal, we wish to encapsulate the vision of “nothing about me without me”.

The journal of *Research Involvement and Engagement* provides a dedicated place for publication of research focused on public involvement and engagement, as well as a forum for discussion of methodological issues that will lead to increased scientific rigour in public involvement and to an enhanced evidence base to underpin best practice internationally.

## Introduction

Welcome to this first edition of a new journal called *Research Involvement and Engagement*. This is a bold and exciting addition to the BioMed Central portfolio of journals. *Research Involvement and Engagement* (or *RIE*) is an interdisciplinary health and social care journal, focussing on patient and public involvement and engagement in research at all stages, including its production, dissemination and implementation. We publish empirical papers and analyses of patient and public involvement and public engagement with research in health and social care, opinion pieces and methodology articles. We encourage critical reflection which enhances our understanding of involvement and engagement. The launch of *RIE* is very timely and reflects the significant increase in interest in patient and public involvement in research internationally and the need to report public involvement in papers, to generate a sound and robust evidence base from which others can draw to develop best practice.

*RIE* is a new type of journal focused on developing a partnership between the public and researchers. We believe collaborative involvement between the public and researchers plays an important role in developing high-quality research, ensuring it is relevant, acceptable and appropriate for patients and the public [1]. Such involvement also reflects a wider intention, of ensuring a democratic accountability for research, much of which is paid for from public monies, whether by taxation or by donation. In addition, public involvement plays an important role in avoiding waste in research by ensuring we ask questions of relevance to patients and measure outcomes of importance to them [2].

Active forms of involvement in research reflect a fundamental paradigm shift in health and social care research, away from paternalism towards partnership, with members of the public collaboratively involved throughout the research process, including as authors and co-authors of papers. This change in the nature of research reflects an international phenomenon, with many countries encouraging the development of closer collaboration between the public and researchers, exemplified by work of groups such as the Health Technology International Patient and Citizen Involvement Group [3].

While much progress has been made, there is still a long way to go before active involvement and co-production of knowledge become the norm in health research internationally. We hope *RIE* will provide an important conduit for the development of the involvement evidence base, which in many ways, it still in its infancy. We hope to contribute to its maturation and greater recognition internationally. Strategic reviews, such as the “Breaking Boundaries Review” commissioned by the Director General Research and Development/Chief Medical Officer (CMO) Department of Health, England, have strengthened the vision of involvement as “a population actively involved in research to improve health and wellbeing for themselves, their family and their communities”. In this future scenario, involvement will become second nature and research without evidence of public involvement would be considered flawed [4]. We see similar trends in other countries and settings, and we hope *RIE* contributes to this paradigm shift by helping to build the evidence base that will contribute to more effective practice. With this mind, we are particularly keen to receive international submissions from countries at the start of their involvement and engagement journey.

We have tried to reflect our values and ways of working in the way that *RIE* has been established. *RIE* is online and open-access, to ensure that it is available to patients, to the public and to researchers alike, anywhere in the world. Open-access publishing offers the opportunity for widening access to papers, to a deliberative discussion internationally, helping nurture the partnership between the public and researchers. The journal will be co-produced by all key stakeholders, including patients, policymakers, academics and funders. *RIE* has a unique governance structure, which includes patients on the editorial board and a patient as one of the two joint editors. *RIE* operates using an open peer review system, where the reviewers’ names are included on the peer review reports. In addition, if the article is published, the named reports are published online alongside the article as part of a “pre-publication history”. All previous versions of the manuscript and all author responses to the reviewers are also available to readers.

Moreover, because of our editorial commitment to co-production on knowledge and the multi-disciplinary nature of the range of topics we can cover, patients, members of the public and researchers will benefit from having the journal as a shared resource for exchanging ideas and experiences, as well as outcomes and findings.

All articles published by *RIE* are made freely and permanently accessible online immediately upon publication, without subscription charges or registration barriers. It is a fundamental principle of this journal that the knowledge we gain from patient involvement and engagement should made available to all, freely, including of course patients and the public.

Authors of articles published in *RIE* are the copyright holders of their articles and have granted to any third party, in advance and in perpetuity, the right to use, reproduce or disseminate the article, according to the BioMed Central copyright and license agreement [5].

We are excited to publish the initial set of articles and commentaries which cover a broad range of areas of importance in patient and public. The key paper by Crowe and colleagues demonstrates the importance of including the public in the prioritisation of research [6], while Mitchell et al.’s article on biobanking from the patient perspective is very timely as interest in defining the patient or public role in biobanking becomes increasingly important [7]. Our commentaries highlight key areas of thinking in relation to patient and public involvement [8, 9]. We look forward to publishing key papers and commentaries now in the *RIE* pipeline.

## Conclusion

We are very excited about the launch of *Research Involvement and Engagement*. We invite you to submit your papers to *Research Involvement and Engagement*, the first journal anywhere in the world to specialise in patient and public involvement and engagement.
